# Supplementary material for: Assessment of Excess Mortality in Italy in 2020–2021 as a Function of Selected Macro-Factors
Source: Int J Environ Res Public Health. 2023 Feb 5;20(4):2812. doi: 10.3390/ijerph20042812 (PMC9956038; doi:10.3390/ijerph20042812)
Supplement: Supplementary file 1 [file ijerph-20-02812-s001.zip › ijerph-2161083-supplementary.pdf]

1 Clustering models

Model	Σ_k	β_k	Nb. of variance parameters
$\mathrm{DFM}_{[\Sigma_k\beta_k]}$	Free	Free	$(K-1)(p-K/2)+K^2(K-1)/2+K$
$\mathrm{DFM}_{[\Sigma_k\beta]}$	Free	Common	$(K-1)(p-K/2)+K^2(K-1)/2+1$
$\mathrm{DFM}_{[\Sigma\beta]}$	Common	Common	$(K-1)(p-K/2)+K(K-1)/2+1$
$\mathrm{DFM}_{[\alpha_{kj}\beta_k]}$	Diagonal	Free	$(K-1)(p-K/2)+K^2$
$\mathrm{DFM}_{[\alpha_{kj}\beta]}$	Diagonal	Common	$(K-1)(p-K/2)+K(K-1)+1$
$\mathrm{DFM}_{[\alpha_k\beta_k]}$	Spherical	Free	$(K-1)(K-1)(p-K/2)+2K$
$\mathrm{DFM}_{[\alpha_k\beta]}$	Spherical	Common	$(K-1)(p-K/2)+K+1$
$\mathrm{DFM}_{[\alpha_j\beta_k]}$	Diagonal and Common	Free	$(K-1)(p-K/2)+(K-1)+K$
$\mathrm{DFM}_{[\alpha_j\beta]}$	Diagonal and Common	Common	$(K-1)(p-K/2)+(K-1)+1$
$\mathrm{DFM}_{[\alpha\beta_k]}$	Spherical and Common	Free	$(K-1)(p-K/2)+K+1$
$\mathrm{DFM}_{[\alpha\beta]}$	Spherical and Common	Common	$(K-1)(p-K/2)+2$

Table S1: Available models in the DFM family and number of free parameters in covariance matrices when $d = K - 1$ for the DFM models.

Models	Number of Basis					
	10	11	12	13	14	15
$\mathrm{DFM}_{[\Sigma_k\beta]}$	-323303.8	-480759.8	-9053.2	-26984.5	-11771.7	-12726.4
$\mathrm{DFM}_{[\Sigma\beta]}$	-286856.4	-492283.9	-571538.8	-7417.0	-8656.8	-10228.9
$\mathrm{DFM}_{[\alpha_{kj}\beta]}$	-366203.6	-399665.3	**-6638.4**	-7268.5	-9461.8	-9981.4
$\mathrm{DFM}_{[\alpha_k\beta]}$	-322895.7	-623083.7	-213880.9	-7450.7	-8256.8	-9291.4
$\mathrm{DFM}_{[\alpha_j\beta]}$	-303329.6	-461382.7	-644917.0	-7419.7	-9501.4	-56993.3
$\mathrm{DFM}_{[\alpha\beta]}$	-326959.8	-463562.6	-514949.6	-8706.5	-778339.4	-864611.5

Table S2: Models of the DFM tested on the excess mortality data. BIC values are shown in the following setting: 4 clusters, starting values obtained through k-means algorithm and different number of basis. Only models that reached a satisfactory convergence are reported.

2 Correlation between variables

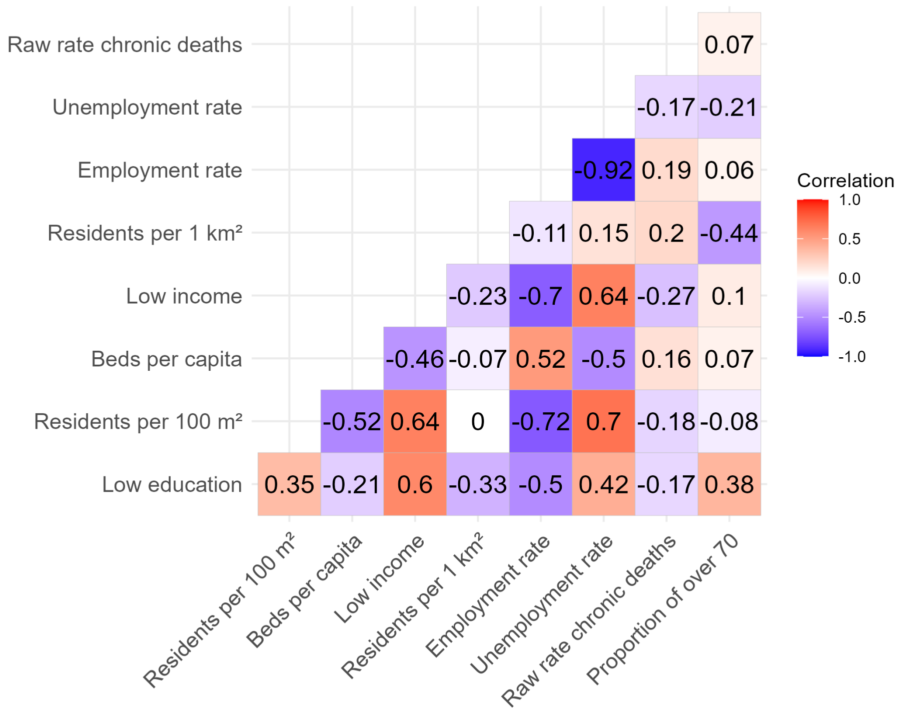

Figure S1: Correlation matrix between variables.

3 Covid timeline in Italy

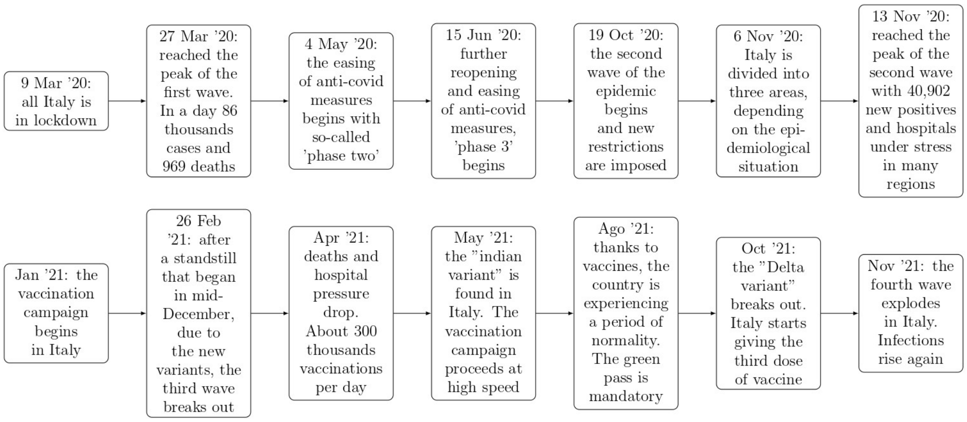

Figure S2: Covid timeline in Italy in 2020 and 2021

4 Statistical models

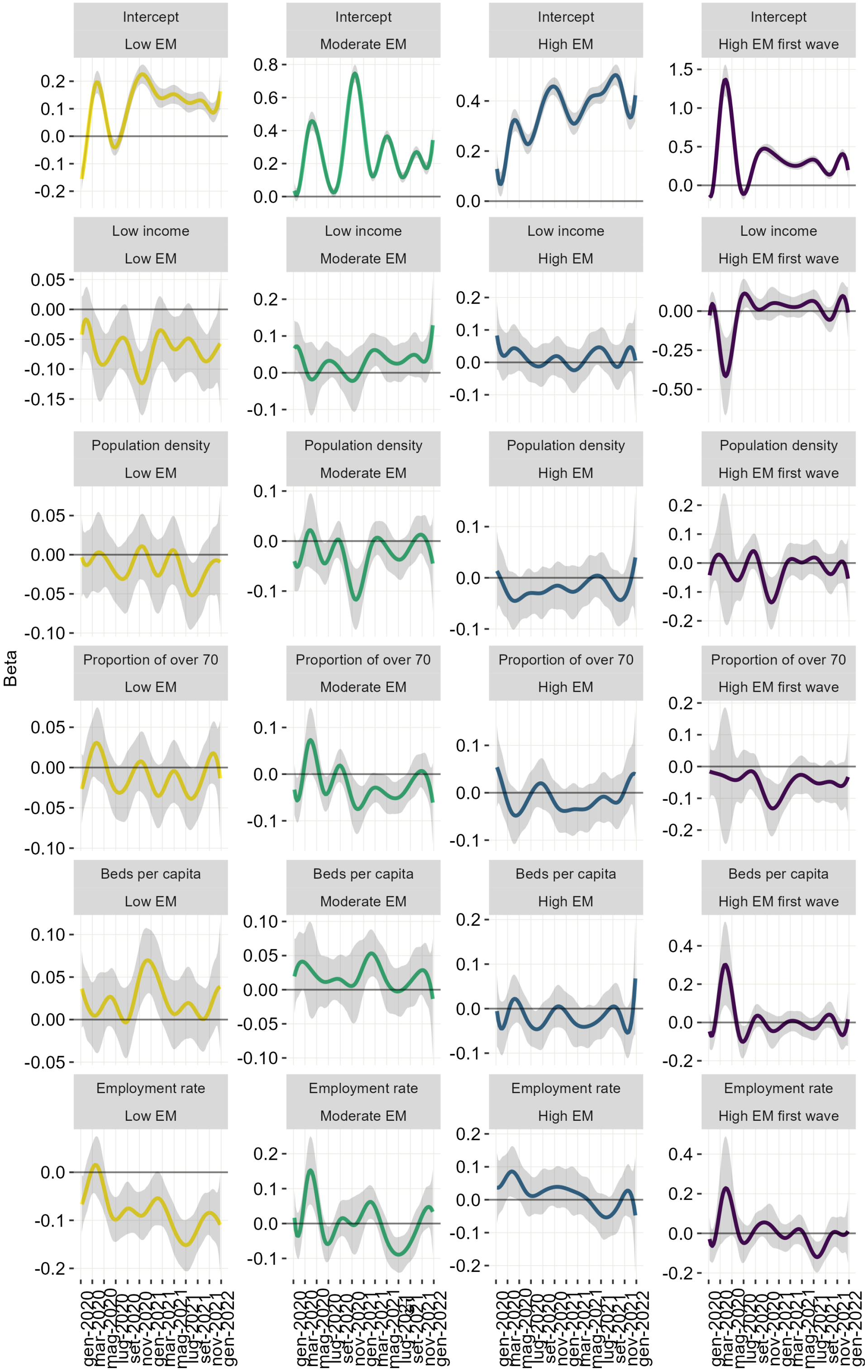

Figure S3: Estimated functional coefficients. The colour of the curves follows the colours of the clusters, namely, yellow - Cluster 1, green - Cluster 2, blue - Cluster 3, and violet - Cluster 4.

Four graphs were constructed for each model to assess the quality of the predictions. The four graphs are:

(a): a scatter plot comparing predicted and actual values of EM. To this was added in red the bisector of the first and third quadrant, indicating the ideal position that the values should have;

(b): a scatter plot comparing predicted EM and the residuals of the model (computed as difference of the actual and the predicted values of the EM). The two horizontal blue dashed lines are placed at $\pm 1.96 \cdot \sigma_{residuals}$, where 1.96 chosen to obtain a 95% confidence interval and $\sigma_{residuals}$ represents the standard deviation of the residuals.

(c): The distribution of residuals, represented with a histogram. The graph was overlaid with a vertical line at zero and the curve of the density of a normal with mean 0 and variance equal to the $\sigma_{residuals}$;

(d): The graphical representation of the quintiles of the residuals distribution, or Q-Q plot. In this type of plot, the theoretical quantiles of a normal distribution are plotted on the horizontal axis. By contrast, the quantiles of standardized residuals are plotted on the vertical axis.

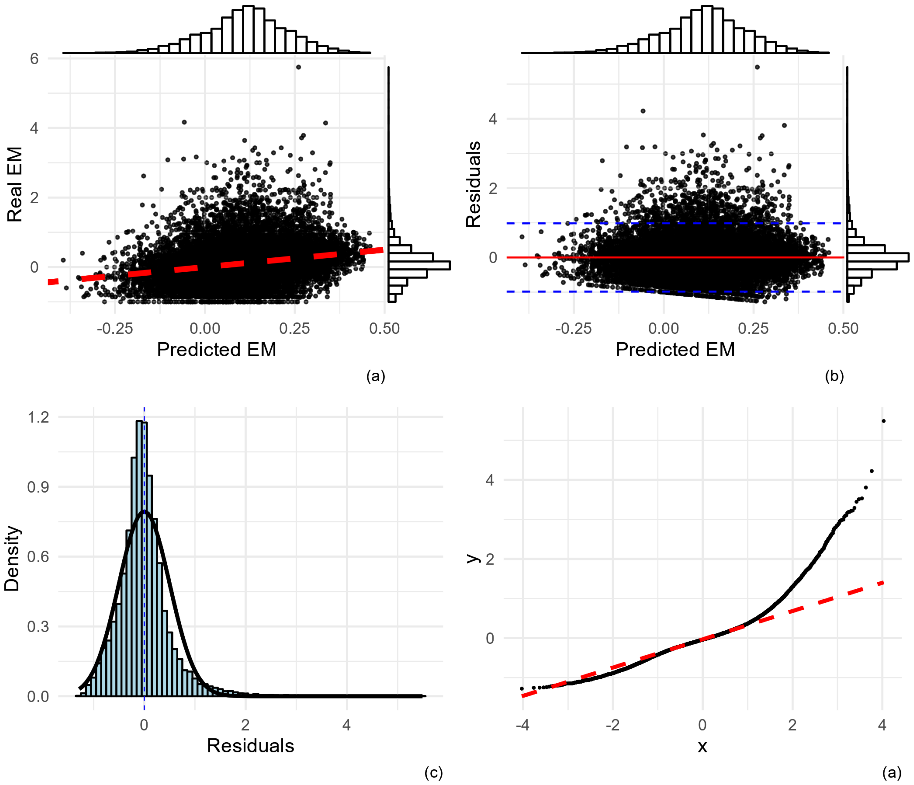

Figure S4: Diagnostic graphs for model in cluster 1

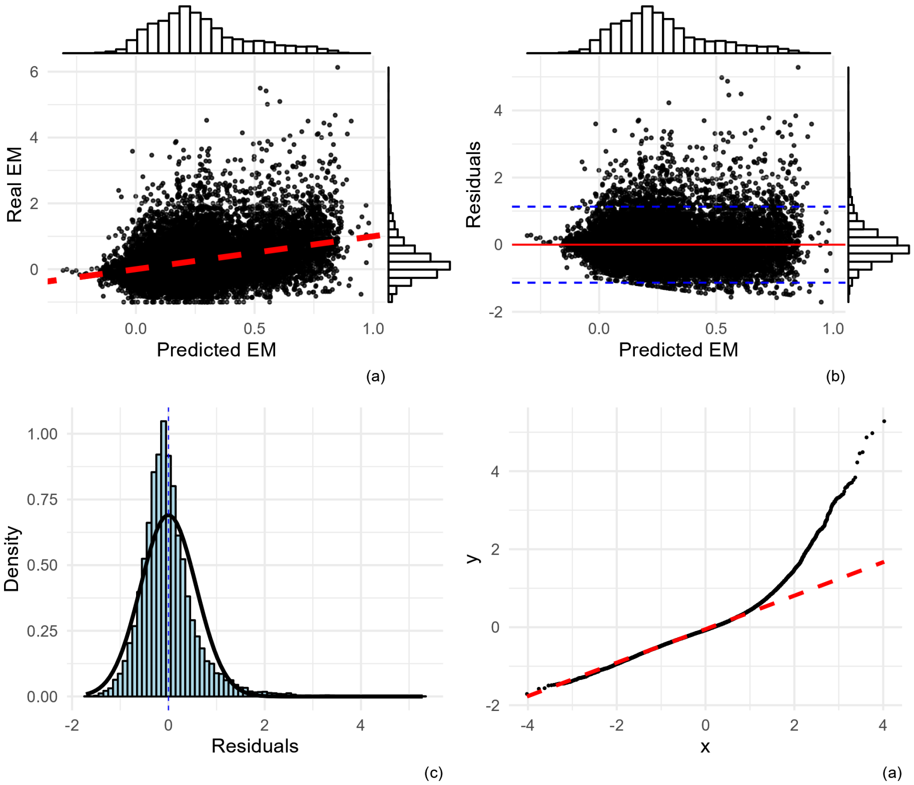

Figure S5: Diagnostic graphs for model in cluster 2

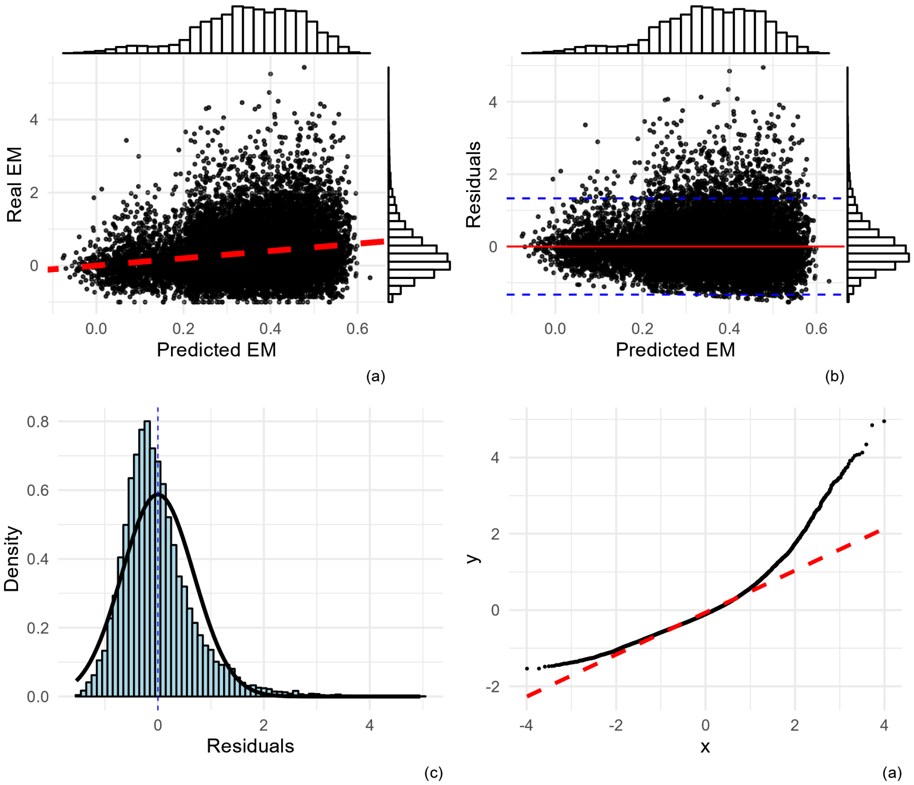

Figure S6: Diagnostic graphs for model in cluster 3

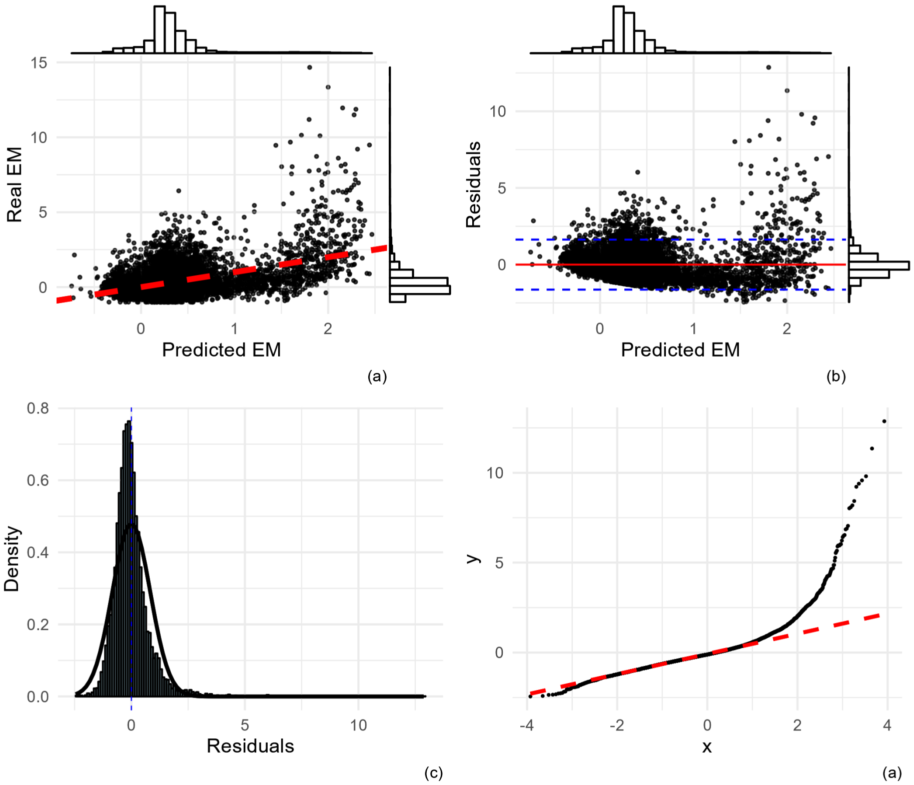

Figure S7: Diagnostic graphs for model in cluster 4

	RMSE	Mean	Standard Deviation
Cluster 1	0.50	0.11	4.88
Cluster 2	0.58	0.27	2.12
Cluster 3	0.68	0.35	1.92
Cluster 4	0.83	0.36	2.32

Table S3: Root mean square error, mean and standard deviation of the four models fitted values.
